# A Disposable Microfluidic Virus Concentration Device Based on Evaporation and Interfacial Tension

**DOI:** 10.3390/diagnostics3010155

**Published:** 2013-02-28

**Authors:** Jane Yuqian Zhang, Madhumita Mahalanabis, Lena Liu, Jessie Chang, Nira R. Pollock, Catherine M. Klapperich

**Affiliations:** 1 Department of Biomedical Engineering, Boston University, 44 Cummington Mall, Boston, MA 02215, USA; E-Mails: janeyqz@gmail.com (J.Y.Z.); smahalanabis.bu@gmail.com (M.M.); lenais@bu.edu (L.L.), chillair@gmail.com (J.C.); 2 Division of Infectious Diseases, Beth Israel Deaconess Medical Center and Department of Lab Medicine, Boston Children’s Hospital, 300 Longwood Ave, Boston, MA 02115, USA; E-Mail: nira.pollock@childrens.harvard.edu

**Keywords:** microfluidic, sample preparation, virus, point-of-care diagnostics, influenza, PCR

## Abstract

We report a disposable and highly effective polymeric microfluidic viral sample concentration device capable of increasing the concentration of virus in a human nasopharyngeal specimen more than one order of magnitude in less than 30 min without the use of a centrifuge. The device is fabricated using 3D maskless xurography method using commercially available polymeric materials, which require no cleanroom operations. The disposable components can be fabricated and assembled in five minutes. The device can concentrate a few milliliters (mL) of influenza virus in solution from tissue culture or clinical nasopharyngeal swab specimens, via reduction of the fluid volume, to tens of microliters μL). The performance of the device was evaluated by nucleic acid extraction from the concentrated samples, followed by a real-time quantitative polymerase chain reaction (qRT-PCR). The viral RNA concentration in each sample was increased on average over 10-fold for both cultured and patient specimens compared to the starting samples, with recovery efficiencies above 60% for all input concentrations. Highly concentrated samples in small fluid volumes can increase the downstream process speed of on-chip nucleic acid extraction, and result in improvements in the sensitivity of many diagnostic platforms that interrogate small sample volumes.

## 1. Introduction

Point-of-care (POC) diagnostics are often small in order to enhance portability, and are thus limited in their ability to process large input samples. For clinically relevant pathogen loads to be detectable in the relatively small volumes of fluid manipulated in microfluidic chips, sample concentration or enrichment is often required. Enrichment involves letting the target bacteria multiply in the sample for a period of time before testing, and in some cases can add hours to a test turn around time. Concentration is easily accomplished in a laboratory setting with a centrifuge. However, as the size and density of the pathogen decreases, the centrifuge becomes larger, more expensive, and less portable. For example, for concentrating viral samples, an ultracentrifuge is often required. Centrifugation is hard to realize at the POC, where devices are required to be simple to fabricate, low cost to manufacture, disposable, flexible to integrate with other microfluidic modules, easy to operate, and relatively rapid. 

Recent efforts to capture and concentrate biological samples in microfluidics are aimed at increasing efficiency and portability, while reducing time and cost of the assay. Existing concentration methods utilize electrodynamics [1,2,3,4,5], filtration [6,7,8], immunomagnetic capture [8,9,10,11], or evaporation [12,13,14,15,16,17,18]. Electrodynamic methods involve manipulating sample flow using an electric field induced between two electrodes. Negatively charged cell membranes or viral envelopes can be manipulated to move towards the positively charged region. Micro-filters can be made out of silicon, glass [6,7], or microporous polymers [8], to allow fluid to pass but pathogens larger than the filter to be trapped. In immunomagnetic capture, pathogens bind to paramagnetic beads via specific antibodies conjugated on the surface. Reichmuth *et al.* combined the advantages of electrophoresis and microfiltration through a porous polyacrylamide gel to concentrate and detect antibody-influenza complexes [8]. Lien *et al.* used a rotary microfluidic device to rapidly mix, purify, and concentrate dengue virus bound to paramagnetic beads functionalized with specific antibodies [11]. Potential limitations of relying on immunocapture and electrophoresis include the need for reagents specific to each target virus type, potential disruption of the intrinsic particle surface properties, and accessories to generate the magnetic and electric fields, which reduce the portability. 

Concentration by microfluidic evaporation is another option that may provide compact and simple operation at the POC. An evaporator designed by Walker and Beebe featured a single channel with passive air evaporation at the outlet to drive sample flow and concentration gradient formation near the outlet [16]. Sharma *et al.* built a continuous-flow enhanced evaporator to concentrate bovine serum albumin in a large volume of water [14]. Leng *et al.* demonstrated convective evaporation with active gas flow to drive fluid flow and salt concentration in a straight channel to study phase transitions during crystallization [19]. In past work*,* we developed a microfluidic evaporation system that exploits surface tension driven meniscus dragging to concentrate dilute bacteria in buffer samples from hundreds of microliters down to sub-microliter volumes in 15 min or less, with greater than 90% recovery efficiency [18]. 

These evaporation devices have been used to concentrate bacteria, microspheres, proteins, toxins, and electrolytes, covering a range of analyte sizes and surface properties. In comparison, viruses are much smaller than bacteria, but are much larger than biological molecules. Their surface properties make them prone to non-specific adhesion to device surfaces. It is the focus of this paper to exploit the physical forces that govern the motion of virions during surface tension driven meniscus movement induced by convective evaporation in a microfluidic channel to minimize interactions with the device surfaces and concentration time, and maximize concentration factor and recovery efficiency. 

## 2. Theoretical Underpinnings

**Figure 1:**
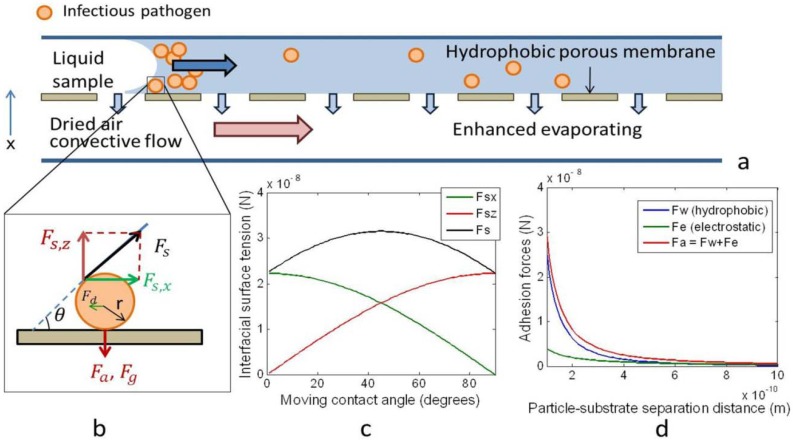
The evaporation microfluidics utilize a mass exchange process driven by the partial pressure gradient of the water vapor across a hydrophobic porous membrane to concentrate particles along the moving fluid meniscus. (**a**) Schematic of the convective evaporation microfluidic device. As convective air flows in the lower layer, liquid sample on the upper layer evaporates through the pores. (**b**) Free body diagram of a single viral particle in the fluid meniscus. These forces include the interfacial surface tension (*F_s_*), adhesion force (*F_a_*), which is proportional to the sum of van der Waal’s and electrostatic forces, Stokes drag force (*F_d_*), gravitational force (*F_g_*), and buoyancy forces (not shown). (**c**) The interfacial surface tension is decomposed in the horizontal (*F_sx_*) and vertical (*F_sz_*) directions. As the moving contact angle increases, *F_sx _*decreases, while *F_sz_* increases. (**d**) The adhesion force between the particle and the substrate (*F_a_*) decreases as their separation distance increases.

The micro-evaporation device described here includes a liquid sample layer and a gas flow layer, between which a hydrophobic porous membrane is sandwiched. As shown in Figure 1, the physical processes in the convective evaporation chip involve mass transport of the fluid (liquid and gas) and of particles (solids) in the fluid. Fluid transport is driven by the partial pressure gradient across the liquid/gas interface at the porous membrane. The rate of fluid mass transport is directly proportional to its vapor pressure in the gaseous mixture. The governing equation for this phenomenon is

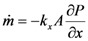
(1)
where 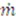 is the rate of evaporation, *k_x_* is the convective mass transfer coefficient, *A* is the area of the exchange surface, 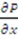 is the pressure gradient from the liquid/gas interface to the convective air flow channel [17]. 

Meanwhile, the pathogens, here viruses, are modeled as sub-micrometer particles suspended in the liquid. As the meniscus moves towards the channel outlet, particles are dragged with the moving meniscus and concentrated. Many competing forces act on the particles, especially on the particles that are close to the channel walls, which determine their locations relative to the fluid (Figure 1). These forces include the interfacial tension, adhesion force (the product of the sum of van der Waal’s and electrostatic forces), Stokes drag force, gravitational force, and buoyancy forces [20,21]. 

The motion of particles in the fluid is strongly affected by the competition between hydrodynamic capillary flow and interfacial tension. Capillary flow of nano- to micro-sized particles during passive evaporation of a sessile drop has been studied extensively. Deegan *et al.* described the differential evaporative flux as the driving force for coffee particles to concentrate at the rim of a pinned contact line, forming a coffee stain [22]. Hu and Larson further defined the phenomenon using PMMA particles that concentrated near the center of a drying octane droplet due to Marangoni effect [23]. Wong *et al.* explored using an evaporating droplet to simultaneously concentrate and separate different sized biological particles at the contact line [21]. This effect is exploited in this paper in the enhanced evaporation platform by creating a moving contact line that rapidly collects and concentrates particles from a relatively large volume of fluid into a desired location and small volume. 

The interfacial tension acting on the solid particles by the fluid is estimated to be

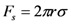
(2)
where *r* is the particle radius, *σ* is the liquid-vapor interfacial tension (0.071 N/m for water), and *θ* is the fluid-substrate moving contact angle. The van der Waal’s force is

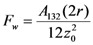
(3)
where *z*_0_ is the particle-substrate separation, *A*_132_ is the Hamakers number for a particle in water (~3 × 10^−20^ J). The electrostatic force is

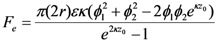
(4)
where *ε* is the dielectric constant of water (7 × 10^−10^ F/m), *κ* is the reciprocal of the Debye length (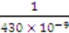) [21], 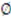 is the zeta potential of the interaction of the particle and the channel surface (here, assuming −40 mV for virus, and −100 mV for Teflon) [21,24,25]. The adhesion force is

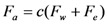
(5)
where *c* is a constant dependent on particle concentration and the statistical possibility of the particles interacting with the channel surface.

Other body forces include buoyancy and gravitational force

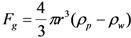
(6)
and Stokes drag force,

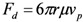
(7)
where *ρ_p_* and *ρ_w_* are the density of the particle and of water, *μ* is the dynamic viscosity of water, and *υ_p_* is the relative velocity of particles in the fluid [20]. The effects of buoyancy, gravity, and Stokes drag on particle motion are small compared to the interfacial tension and adhesion forces, and thus are neglected. From the free body diagram in Figure 1, if surface tension dominates, the particles will be carried with the fluid to the outlet as fluid volume decreases.

The interfacial tension between particle and fluid is proportional to the liquid-vapor surface tension and the particle radius. It can be decomposed into a horizontal (*F_s,x_*) and a vertical (*F_s,z_*) component. For a theoretical particle with a radius of 50 nm, the interfacial tension and its horizontal and vertical components are plotted in Figure 1. One can see that when the surface changes from hydrophilic to hydrophobic, represented by an increase in the moving contact angle, *θ*, *F_s,x_* decreases from 2.25 × 10^−8^ N to 0, but *F_s,z_* increases from 0 to 2.25 × 10^−8^ N.

The interfacial tension is balanced by the adhesive van der Waal’s (*F_v_*) and electrostatic (*F_e_*) forces, which are plotted against the particle-substrate separation distance in Figure 1. Both *F_v_* and *F_e_* increase rapidly when the separation between particles and the device wall decreases to below 0.5 nm. When the separation distance is below 0.1 nm, the adhesive force on one particle would be above 3 × 10^−8^ N, comparable to the interfacial tension. As particle concentration increases, the particles become more closely packed. As a result, the probability for the particles to collide or to get very close to the channel surface increases. This probability is indicated by the empirical coefficient *c*, which would increase the collective adhesive force when the particle concentration is high. Therefore, special attention was paid to reduce surface adhesion by blocking the hydrophobic substrate materials used to make the channels, which included a Teflon membrane and a silicone-based pressure sensitive adhesive (PSA).

**Table 1:** Physical parameters, assumptions, and calculated forces used in particle motion analysis.

Physical meaning	Parameter	Unit	Particles in the analyte		
			*E. coli*	influenza	influenza with surface treatment
particle radius	*r*	m	5.00E−07	5.00E−08	5.00E−08
liquid-vapor surface tension [21]	*σ*	N/m	0.071	0.071	0.071
liquid-substrate contact angle	*θ*		50	50	10
		rad	0.87	0.87	0.17
liquid-particle surface tension	*F_s_*	N	2.23E−07	2.23E−08	2.23E−08
	*F_s,x_*	N	1.43E−07	1.43E−08	2.20E−08
	*F_s,z_*	N	1.71E−07	1.71E−08	3.87E−09
Hamaker constant when particles and substrate are in liquid [21]	*A132*	J	3.00E−20	3.00E−20	3.00E−20
Minimum particle-substrate separation [21]	*Z_0_*	m	4.00E−10	4.00E−10	1.00E−09
van der Waal's force	*F_v_*	N	1.56E−08	1.56E−09	2.50E−10
permitivity of water [21]	*ε*	F/m	7.00E−10	7.00E−10	7.00E−10
reciprocal of Debye Length [21]	*κ*	1/m	2.33E+06	2.33E+06	2.33E+06
zeta potential of particle [21]	*φ_1_*	mV	−40	−40	−40
zeta potential of Teflon [21]	*φ_2_*	mV	−100	−100	−100
electrostatic force	*F_e_*	N	9.87E−09	−5.11E−12	−5.11E−12
Empirical indicator of concentration threshold	*c*		7	11	16

## 3. Materials and Methods

### 3.1. Device Fabrication

**Figure 2:**
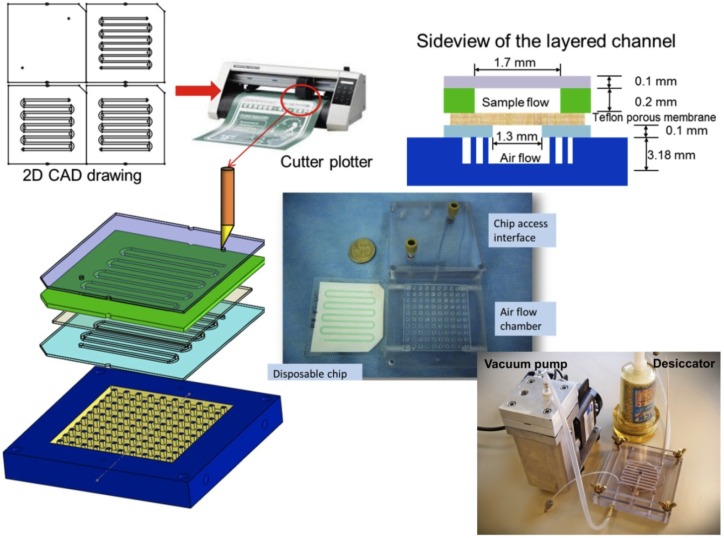
Fabrication and side view of the chip achieved with maskless xurography. Also shown is the chip with air flow chamber and access interface prior to assembly. The entire system with portable vacuum pump and desiccator is shown at the bottom.

The polymeric device included a fluid sample layer, an airflow control layer, a hydrophobic porous membrane layer sandwiched in between, and an air flow chamber (Figure 2). The layers included a silicone pressure sensitive adhesive (PSA) on a polyolefin backing (TempPlate RT Select Optical Film, USA Scientific, Ocala, FL, USA, #2921-7800), a double-sided adhesive (Double Tack^®^, Grafix, Cleveland, OH, USA, #KDT3), a 0.2 μm PTFE laminated membrane filter (Sterlitech, Kent, WA, USA, #PTFE0214225), and a polycarbonate plaque (McMaster Carr, Chicago, IL, USA), respectively. The 2D CAD patterns on the fluid sample and the air flow control layers were drafted in SolidWorks^®^ (SolidWorks, Concord, MA, USA), and were cut with a Graphtec Craft ROBO ProS cutter plotter (Graphtec America, Santa Ana, CA, USA, model: CE5000-40-CRP), with the software ROBO Master Pro (Graphtec America, Santa Ana, CA, USA). The layers were aligned to the air flow chamber, and sealed by applying pressure to the assembly. The air flow chamber had a vacuum connector and three air inlets. On the chip access interface, there were inlets and outlets tapped to connect to a 1/16'', 10-32 PEEK fitting (IDEX Health & Science, Oak Harbor, WA, USA, #F-120x). The finger tight PEEK fitting allowed one to fit in a pipette tip snuggly to dispense the sample and act as a reservoir for larger sample storage during evaporation.

After testing, the double-sided tape was detached and the chip was discarded, while the air flow chamber and chip access interface were sterilized and reused. The entire fabrication process of the disposable, from CAD design to a completed device ready for testing, took less than 5 min. 

### 3.2. Visualizing Concentration with Fluorescent Beads

The device was tested first using fluorescent beads. Standard 100 nm fluorescent polystyrene beads (FluoSpheres^®^ carboxylate-modified microspheres, yellow-green fluorescent (505⁄515), Invitrogen, Carlsbad, CA, USA, #F8803) were used to mimic viral particles to visualize concentration in the device. To operate the concentration device, a vacuum pump was connected to the air outlet, such that air was pumped in from ambient through desiccators to the air flow chamber at a pressure of 100 kPa. 150 μL of fluorescent beads 100× diluted in distilled water was dispensed to fill the channel. To monitor the progress of concentration, a fluorescent image was taken every 3 min using a VersaDoc 400 imaging system (Bio-Rad Laboratories, Hercules, CA, USA), with an LP520 filter and a 3 s exposure time. The evaporation process was stopped at 24 min, when there was approximately 5 μL remaining in the device. The experiment was repeated 3 times. The images were processed with ImageJ (http://rsb.info.nih.gov/ij/) to subtract background consistently across all images. The overall fluorescence from the entire imaging channel and the fluorescence from a 1 mm^2 ^area near the sample outlet was plotted in the same figure against time. 

### 3.3. Viral Culture and Patient Sample Collection

Next, concentration experiments were run using cultured influenza A, followed by experiments using human nasopharyngeal swab samples. The cultured influenza A/PR/8/34 (ATCC, Manassas, VA, USA, #VR-1469^®^) was grown in Madin-Darby canine kidney (MDCK, CCL-34, ATCC, Manassas, VA, USA) cell culture. The virus inoculation and harvest was performed according to the WHO Manual on Animal Influenza Diagnosis and Surveillance [26]. Specifically, MDCK cells were cultured in the Eagle’s Minimum Essential Medium with Earle’s BSS and 2mM L-glutamine (EMEM) with 1.0 mM sodium pyruvate, 0.1 mM nonessential amino acids, 1.5 g/L sodium bicarbonate, 10% fetal bovine serum (FBS), 100 IU/mL penicillin and 100 ug/mL streptomycin, and incubated at 37 °C with 5% CO_2_. Cells were passaged every 3 days or when the flask was 80–90% confluent by detaching with trypsin-EDTA (Invitrogen, Carlsbad, CA, USA, #25200) and transferring enough cells to generate a 90% confluent flask in 3 days. Upon reaching confluence, cells were washed with 1X PBS (Fisher Scientific, Pittsburg, PA, USA, #MT-21-031-CV), and the culture medium was replaced with the viral culture medium (VCM): EMEM with 2 μg/mL TPCK-treated trypsin, 0.6% BSA, 0.025 M HEPES buffer, and 100 IU/mL penicillin/100 μg/mL streptomycin. Frozen influenza virus in media from stock was quickly thawed in 37 °C water bath to inoculate the cells in the flask. The cells were observed daily, until viral infection peaked and cell monolayer was destroyed (approximately 5–7 days). Virus was then harvested from the cell culture supernatant by centrifuging at 4,000 rpm or 2,000× g for 7 min at 4 °C. The supernatant was divided into aliquots of 0.5 mL and stored at −80 °C until use. 

The patient specimens were discarded nasopharyngeal swab (NS) specimens collected at Beth Israel Deaconess Medical Center (BIDMC) during the 2008–2010 flu seasons. This study was reviewed and approved by BIDMC’s institutional review board. The specimens were collected as part of routine clinical care from patients presenting with one or more symptoms consistent with influenza, including fever, myalgia, cough, sore throat, nasal congestion or rhinorrhea (runny nose), headache, malaise, or diarrhea. Subjects were adults and of both genders and diverse racial/ethnic backgrounds. NS specimens were taken using Copan flocked swabs (COPAN, Murrieta, CA, USA). The swab was inserted flat and pushed forward with gentle downward pressure on the lower nasal floor to the posterior wall of the nasopharynx, where it was rotated for a few seconds to collect cellular material. The swab was withdrawn, placed into sterile 1× PBS, and submitted on ice to the BIDMC microbiology laboratory. After routine direct fluorescent antigen (DFA) testing, specimens were stored (1.0 mL aliquots) at −80 °C and subsequently deidentified and transferred to the Klapperich laboratory for further storage at −80 °C. Only DFA-positive samples were used in this study. Before testing, all specimens were routinely quick-thawed from −80 °C in a 37 °C water bath, and serially diluted with 1× PBS. 

### 3.5 Concentration of Influenza Viral Sample with Evaporation

To run both the cultured influenza A and the patient specimens in the chip, the following protocol was used. The device was treated with 7.5% BSA or viral culture medium (VCM) by incubation for 30 min as a blocking step. The blocking solution was removed prior to loading the device. To operate the viral concentration device, a vacuum pump was connected to the air outlet, such that air was pumped in from the ambient air through desiccators to the air flow chamber at a pressure of 100 kPa. The device was heated to 35 °C during evaporation by a conventional hot plate. While the heating speeds up the concentration process, it will proceed in a similar fashion at a slower rate without it. The cultured viral sample was quickly thawed in a 37 °C water bath. The copies/mL for the cultured samples were estimated using qRT-PCR (see Section 3.7). RT-PCR was run, and the C_T_ values obtained were compared to a standard curve made using known concentrations of synthetic plasmid DNA carrying the M1 gene. Separately, plaque assays were run on the same cultured samples so that a semi-quantitative relationship between copies/mL and PFU/mL could be obtained. Using this calculation, a conversion between copies/mL and PFU/mL for the cultured samples was made. Assuming that the infectivity of the patient samples and the cultured samples were similar, the PFU/mL of the patient samples were calculated using the same equation.

All samples were serially diluted to approximately 1 PFU/mL in 1× PBS based on the above calculation of the initial concentration, and stored on ice. Starting from the lowest concentration, 1 mL of sample was pipetted into the inlet and allowed to completely fill the sample flow channel, driven by the negative pressure inside the channel. A pipette tip containing the balance of the sample fit snuggly at the inlet connector as a reservoir. The outlet was sealed until the end of evaporation. Fluid from the sample was continuously evaporated, causing the fluid to deplete from the pipette tip reservoir and the meniscus to travel through the channel from inlet to outlet. Evaporation was stopped by turning off the vacuum pump when the sample volume was reduced to 50 μL. As a control, 50 μL of the original input sample was set aside and was called the “un-concentrated” control. Both the concentrated output sample and the un-concentrated input sample were processed in Qiagen QIAamp viral RNA mini kit (Qiagen, Valencia, CA, USA, 52904) to extract the viral RNA. The extracted sample was amplified using qRT-PCR as described in Section 3.7. PBS was the negative control for both processes. From the qRT-PCR, the viral RNA recovery efficiency was calculated as the ratio of RNA copies in the output over the input viral samples (Equation (8)). The concentration factor was the ratio of RNA concentration in the output over the input viral samples (Equation (9)). It is worth noting that the recovery includes RNA from all viral particles, infectious or not, and is not indicative of the recovery of infectivity. 


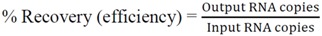
(8)


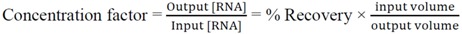
(9)

### 3.6. RNA Extraction

50 μL of both the input (control) and output influenza samples were diluted to 140 µL and were extracted using a QIAamp Viral RNA Mini kit (Qiagen, Valencia, CA, USA, 52904) as per the manufacturer’s instructions. The extracted RNA was eluted in 60 μL of nuclease free water and stored at −80 °C before further analysis.

### 3.7. Quantification Using qRT-PCR

The extracted viral RNA was quantified using qRT-PCR. The CDC protocol for influenza A(H1N1) [28] was followed, but without the human RNase P control. The Invitrogen (Carlsbad, CA, USA) SuperScript^®^ III Platinum^®^ One-Step qRT-PCR Kit w/ROX (11745-100) was used, and the primers/probe were purchased from Biosearch Technologies’ (Novato, CA, USA) Swine Flu Panel (Universal Influenza A, Forward Primer: GACCRATCCTGTCACCTCTGAC, Reverse Primer: AGGGCATTYTGGACAAAKCGTCTA, and Probe: TGCAGTCCTCGCTCACTGGGCACG). The qRT-PCR was completed in an ABI 7300 PCR machine (Applied Biosystems, Foster City, CA, USA). Each reaction consisted of 12.5 μL TaqMan Universal PCR Master Mix 2×, 0.5 μL SuperScript III RT/Platinum Tag Mix, 0.5 μL of 40 μmol/L forward and reverse primers, and 0.5 μL 10 μmol/L dual-labeled fluorescent probe and was adjusted to a total volume of 20 μL with nuclease-free water. Finally, 5 μL of isolated RNA per well was added to a total reaction volume of 25 μL. The qRT-PCR reaction was performed in 3 stages, including reverse transcription for 30 min at 50 °C, Taq inhibitor inactivation for 2 min at 95 °C, and 45 cycles of a 2-step program: denaturation at 95 °C for 15 s, then annealing and extension at 55 °C for 30 s. The fluorescence was read at the completion of the 55 °C step. For each experiment, a no-template reaction (nuclease-free water) was included as a negative control. Each RNA sample was tested in triplicate, and the mean values were calculated. Triplicate values varied by no more than 10% from the mean. We used the standard curve absolute quantification technique to quantify copy number of cDNA in the reaction. A standard curve was generated using a 10-fold dilution series of plasmids containing the M1 gene synthesized by SeqWright (Houston, TX, USA). The number of PCR cycles required for the threshold detection of the fluorescence signal (C_T_) was determined for each sample. C_T_ values of the standard samples were determined and plotted against the log amount of standard. C_T_ values of the unknown samples were then compared with the standard curve to determine the amount of target in the unknown sample. Standard curves from each experiment were compared to ensure accurate, precise, and reproducible results. 

## 4. Results and Discussion

### 4.1 Fluorescent Beads Quantification

**Figure 3:**
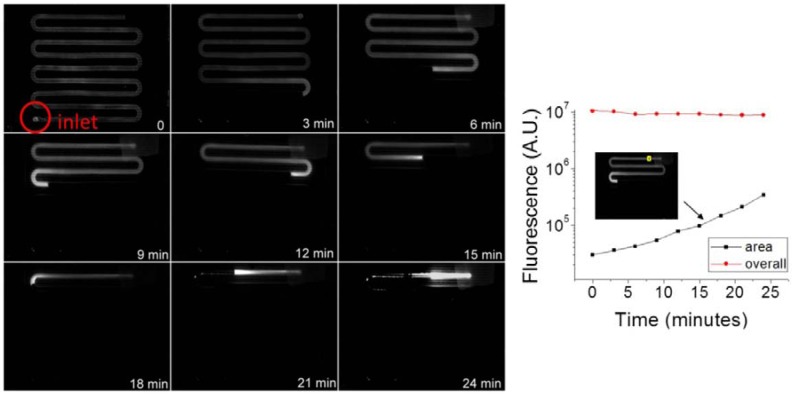
Concentration of 100nm Fluorescent beads in the evaporator. **Left**: representative fluorescent images of the channel over time. **Right**: fluorescence inside the entire channel and in a 1mm^2^ area near the outlet over time.

We used 100 nm fluorescent beads to mimic and visualize the movement of the virus inside the channel in a qualitative fashion, and to demonstrate functionality of the device for particles in the influenza virus size range (80–120 nm in diameter). Particular attention was paid to the intensity and length of the fluorescent region in Figure 3. Increased intensity indicated increased concentration of beads, while the overall length of the fluorescent region indicated movement of the meniscus. In Figure 3, the overall fluorescence of the beads inside the entire channel decreased by 15% from the beginning to the end of evaporation, as the length of the fluorescent region shortened, indicating losses due to photobleaching and particle retention on the channel walls. Loss of particles to the channel walls was seen as the residue fluorescence left behind the moving meniscus, as the local particle concentration increased, as seen at 21 and 24 min. The fluorescence near the moving meniscus increased earlier than in the rest of the fluid, indicating particles were collected at and concentrated by the moving meniscus first. One can also deduce from the location of the meniscus that the fluid evaporation speed was not uniform throughout the 24 min. This result was expected as the evaporation contact area between the fluid and air decreases as the fluid volume decreases. In Figure 3, the fluorescence in a 1 mm^2^ area near the outlet increased exponentially over time, indicating particles were concentrated as they moved towards the outlet. Repeated tests (n ≥ 3) showed that concentration of 100 nm particles was achieved successfully in the evaporation device, with uneven meniscus movement speed, and 15% loss of particles. 

Nominally, one would expect 100 nm particles to pass through a 0.2 µm filter. However, this was not the case with our device. The combination of flow lateral to the pores (rather than perpendicular) and the hydrophobic nature of the filter retained all of the observed beads on the fluid side of the filter. The major cause of the loss of particles is the increased particle interaction with the channel walls at higher concentrations and slower meniscus speeds. If the non-specific van der Waal’s interaction between the particles and the wall dominated the interfacial tension between the particles and the fluid at very small particle-wall separations, there was a greater possibility for particles to adhere near the outlet rather than the inlet. Therefore, ways to reduce such loss would be to smooth the channel walls, or to reduce non-specific interactions between the particles and the channel walls.

### 4.2. Concentration of Influenza Samples

**Figure 4:**
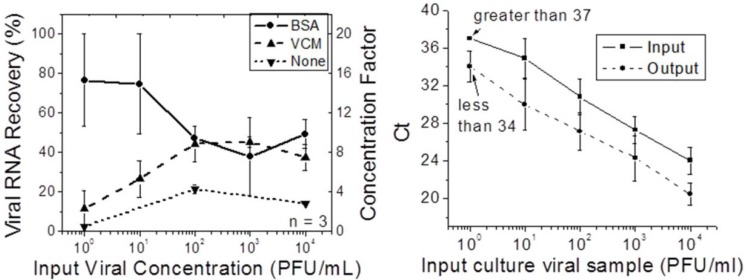
**Left**: Viral RNA recovery and concentration factor for different input (cultured) viral concentrations after chip surface treatment by BSA, viral culture medium (VCM), compared to non-treated chips. **Right**: Comparison of C_T_ before and after concentration for cultured viral samples.

After the bead experiments, experiments using cultured influenza A were run. Different blocking methodologies were tested using the cultured viruses. In Figure 4, the viral RNA recovery and concentration factors were plotted against different input concentrations of cultured viral samples. Both the BSA and VCM coated chips recovered more viral RNA than non-treated chips, confirming the necessity to coat the chip surface prior to sample concentration. The VCM was equally as effective as BSA for input viral concentrations greater than 10^2^ PFU/mL, but less effective than BSA for lower viral input concentrations. This result can be explained by competitive binding affinity of BSA, virus, and VCM to the channel surface in decreasing order. Finally, the amount of viral RNA recovery was related to the input concentrations. Recovery was higher for higher input concentrations than for lower input concentrations for VCM-treated chips. For the BSA-treated chips, the effect was reversed. For lower input concentrations, BSA-treated chips had an average recovery of nearly 80%. The output sample was 16 times more concentrated than the input sample. This result was consistent with the results of the experiments using fluorescent beads, as lower input concentrations of beads resulted in less loss to the walls in the first stages of the concentration process. As a result, BSA was selected and used as the blocking agent in later tests. 

The lowest input concentration (approximately 1 PFU/mL) was at the lower limit of detection of our qRT-PCR assay, with a C_T_ value higher than 37. The concentrated sample had a C_T_ less than 34, indicating about one order of magnitude concentration of the input sample and a change from a borderline C_T_ value to a value reliably lower than the assay’s limit of detection. 

**Figure 5:**
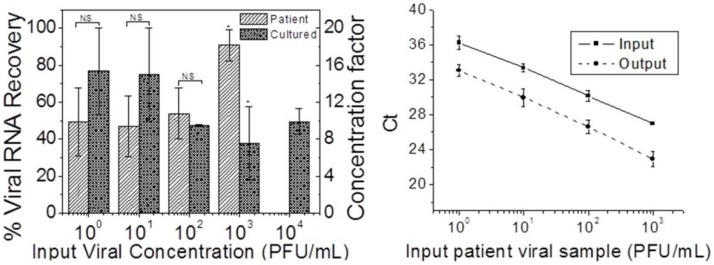
**Left**: Viral RNA recovery and concentration factor for serial dilutions of both patient and cultured influenza samples. Both input and output samples from the concentrator are processed with the QIAamp Viral RNA mini kit. Cultured: n = 3. Patient: n ≥ 3 (for all except the undiluted specimen). NS: not significant; * P < 0.05; two sample unpaired two-tailed Student’s *t*-test. **Right**: Comparison of C_T_ before and after concentration for patient nasal pharyngeal fluid viral samples.

The evaporation concentration procedure was then applied to the clinical nasopharyngeal swab specimens. Viral RNA recovery and concentration factors were compared to those from the cultured virus samples, and are plotted in Figure 5. The C_T _values for patient samples before and after concentration were also plotted in Figure 5. Taking into account the large standard deviation in the cultured samples at low input concentrations, the difference in viral RNA recovery between cultured and patient samples for input concentrations lower than 10^3^ PFU/mL was not statistically significant (two sample unpaired student’s t-test two-tailed p > 0.05). For the 10^3^ PFU/mL input samples, the recovery from the patient samples was significantly higher than from culture samples (p < 0.05). The lower viral RNA recovery from culture samples could be due to higher salt and cell debris content compared to the patient samples. As evaporation progressed, the salts crystallized, and mixed with the debris to form a porous solid plug near the outlet of the concentration chip. Such a plug partially occluded the channel and prevented retrieval of all of the specimens from the outlet, contributing to the lowered recovery efficiency. The standard deviation of the recovery efficiency decreased as the input concentration increased, indicating more consistent performance for higher viral input concentrations. The average RNA recovery efficiency was 60.3% (±20.5%) for patient respiratory samples and 57.3% (±16.5%) for cultured samples, with close to 80% efficiencies for the lowest input concentrations. The average concentration factor was 11.5 (±3.3) for both patient and cultured samples using current methods. These results imply that if the volume ratio between the input (1 mL) and the output (50 µL) sample was further increased (higher than 20), the concentration factor achieved could be higher. In Figure 5, the C_T _values for the concentrated output were consistently lower than those for the un-concentrated input by three or more for all input concentrations, indicating consistent performance of the device. For example, for the 1 PFU/mL input concentration, the Ct was greater than 36, below the limit of detection of our qRT-PCR assay. After concentration, the C_T_ was reduced to 33, giving a conclusive diagnostic result, and would result in a reduction of false negatives at these low input concentrations. 

## 5. Conclusions

We have developed a portable, disposable, simple to fabricate, and highly effective polymeric microfluidic viral sample concentration device that could be used to increase the speed and sensitivity of molecular diagnostics at the POC. We have demonstrated the utility of this device for samples containing variable concentrations of influenza A virus, including both simulated samples spiked with cultured virus and clinical nasopharyngeal swab specimens. Compared with current off-chip concentration methods such as ultracentrifugation and PEG precipitation, this method replaces centrifugation with a disposable chip. Fluorescent polystyrene beads were used as a proof of concept, showing efficient concentration inside the channel and some loss to the channel surface due to surface roughness and non-specific interactions. The non-specific binding of cultured influenza A virus to the channel surfaces was characterized, and several blocking agents were tried. 7.5% BSA was found to be the most effective blocking agent and thus was used in all subsequent viral concentration tests. 

The concentration efficiency from both simulated (spiked) and patient nasopharyngeal swab specimens was characterized by RNA extraction of concentrated and unconcentrated viral samples by Qiagen kit and qRT-PCR. The viral RNA recovery was greater than 60% for both cultured and patient influenza samples, with concentration factors over 12. Finally, the production of a highly concentrated sample in a small fluid volume improved the clinical sensitivity of our diagnostic assay. 

Although testing was focused on influenza virus, samples including a variety of other viruses and bacteria could theoretically be concentrated using this chip. There is potential to integrate this device with downstream on-chip sample preparation and amplification/detection platforms, to achieve more sensitive point-of-care detection of infectious diseases. The main benefit is the reduction in the need for off-chip sample manipulation. Furthermore, there are opportunities to scale up current fabrication methods based on the development of simple lamination technology to further bring down costs. 

A limitation of the evaporation concentrator in its current form is the indiscriminative concentration of the entire content of the input sample. For example, all the proteins, cellular debris, and salts are concentrated and moved by the meniscus towards the outlet of the evaporator. This resulted in decreased concentration efficiency overall, but was suitable for nasopharyngeal swab samples, which contain relatively small amounts of cell debris. To use the evaporation concentrator for other bodily fluids, such as blood, additional steps must be taken to lyse and filter the blood cells and to purify the virus away from proteins and salts. This could be achieved by microscale dialysis, ongoing work in our laboratory. For now, however, the described device is suitable for relatively uncomplicated samples containing viruses and/or bacteria, such as other swab samples (e.g., vaginal), saliva, and cerebrospinal fluid without extensive modifications. Since the concentration technique appears not to lyse the concentrated particles, it is useful in conjunction with diagnostic techniques that require whole particles. Further, the relative simplicity of the concentration method should make it attractive as a front-end sample preparation step for a number of chip-based assays in development. 
